# Loxoprofen-induced interstitial pneumonia: a case report

**DOI:** 10.1186/s13256-016-0919-2

**Published:** 2016-05-25

**Authors:** Motoyasu Kato, Shinichi Sasaki, Yasuhito Sekimoto, Naoko Arano, Hitomi Jo, Kentaro Suina, Sachiko Kuriyama, Keiko Muraki, Osamu Nagashima, Yasuko Yoshioka, Shigeru Tominaga, Kazuhisa Takahashi

**Affiliations:** 1 Department of Respiratory Medicine, Juntendo University Urayasu Hospital, 2-1-1 Tomioka, Urayasu, Chiba 279-0021 Japan; 2 Department of Respiratory Medicine, Juntendo University Graduate School of Medicine, Tokyo, Japan

**Keywords:** Drug-induced lung disease, Loxoprofen, Nonsteroidal anti-inflammatory drug, Organized pneumonia, Steroid

## Abstract

**Background:**

Loxoprofen is a nonsteroidal anti-inflammatory drug used in the treatment of many diseases. However, there are no case reports about loxoprofen-induced pneumonia. We have encountered a rare case of loxoprofen-induced pneumonia.

**Case presentation:**

We report the case of a 71-year-old Japanese woman who was initially treated with loxoprofen for fever. She was admitted to our hospital because of worsening of her symptoms, including fever and dyspnea. Her symptoms improved after treatment with ceftriaxone. Seven days after admission, she again developed high fever. She was again treated with loxoprofen and levofloxacin. However, acute respiratory failure developed after initiation of loxoprofen treatment. Chest computed tomography showed peribronchovascular consolidation. She was diagnosed with loxoprofen-induced pneumonia for which she was administered steroids. After treatment, her dyspnea and radiological findings improved.

**Conclusions:**

The findings in this case report reveal an association between treatment with a nonsteroidal anti-inflammatory drug and pneumonia. This rare case was diagnosed after accidental retreatment with loxoprofen. This is the first report of loxoprofen-induced pneumonia.

## Background

Loxoprofen is a nonsteroidal anti-inflammatory drug (NSAID) that alleviates inflammation and pain by nonselective inhibition of the cyclooxygenase pathway. Many clinicians worldwide routinely administer loxoprofen to patients with cold symptoms and acute upper respiratory inflammation. The major side effects of loxoprofen include gastric inflammation, ulcers, and renal dysfunction. However, development of interstitial pneumonia is a very rare side effect of loxoprofen. Here we report the first case of interstitial pneumonia induced by loxoprofen.

## Case presentation

**Fig. 1:**
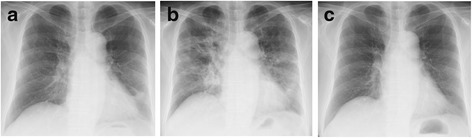
Chest X-ray images. **a** Chest X-ray on admission. **b** Chest X-ray at the time of worsening of respiratory failure. **c** Chest X-ray after steroid treatment

**Fig. 2:**
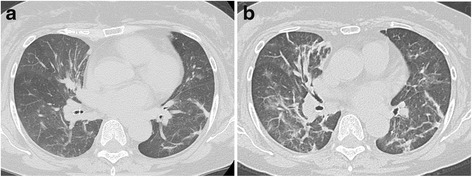
Chest computed tomography images. **a** Chest computed tomography on admission. **b** Chest computed tomography at the time of worsening of respiratory failure

A 71-year-old Japanese woman presented to our hospital with high fever and severe cough, and was admitted for bacterial pneumonia. Before her admission, she had developed fever and cough, and had received treatment with the anti-inflammatory drug loxoprofen. However, her symptoms deteriorated. Her initial vital signs on admission were as follows: temperature, 37.8 °C; respiratory rate, 18 breaths/minute; and oxygen saturation (SpO_2_) on room air, 94 %. A physical examination revealed fine crackles in both her lower lung fields. Her laboratory test values were as follows: white blood cell (WBC) count, 6000/μL; neutrophil count, 3780/μL; serum lactate dehydrogenase (LDH) level, 230 IU/L (normal, 119 to 229 IU/L); and serum C-reactive protein (CRP) level, 6.3 mg/dL (normal, <0.3 mg/dL). A chest radiograph (Fig. 1a) showed reticular shadows in both her lower lung fields. Chest computed tomography (CT; Fig. 2a) showed consolidation. A sputum Gram stain revealed only normal bacterial flora. *Mycoplasma* antigen was absent. Urinary antigen tests for *Legionella* and *Streptococcus pneumoniae* also yielded negative results.

She was treated with ceftriaxone (2.0 g/day) for 7 days. She was retreated with loxoprofen a few times when she had high fever and pain. After these treatments, although her symptoms resolved almost completely, she still had a slight fever. Chest radiograph findings showed improvement of reticular shadows in almost all her lung fields. However, 7 days after initiation of ceftriaxone treatment, she again developed high fever. She was treated with loxoprofen to alleviate her fever, and the antibiotic was changed from ceftriaxone to levofloxacin. After 3 days, she had intermittent high fever, severe cough, and dyspnea, which gradually worsened. Her initial vital signs at this time were as follows: temperature, 39.0 °C; respiratory rate, 24 breaths/minute; and SpO_2_ on room air, 88 %. A physical examination revealed fine crackles in both her lower lung fields. Her laboratory test values were as follows: WBC count, 10,600/μL with a left shift; neutrophil count, 9110/μL; serum LDH level, 255 IU/L; serum CRP level, 14.8 mg/dL; Krebs von den Lungen-6 (KL-6) level, 320 IU/L (normal, <500 IU/L); surfactant protein-D (SP-D) level, 140 ng/mL (normal, <110 ng/mL); and plasma (1→3) beta-D-glucan level, 13 pg/dL (normal, <20 pg/mL). Arterial blood gas values obtained on 3 L/minute oxygen delivered via nasal cannula were as follows: pH, 7.45; partial pressure of oxygen in arterial blood (PaO_2_), 58 Torr; partial pressure of carbon dioxide in arterial blood (PaCO_2_), 36 Torr; and bicarbonate level, 27 mg/dL. A chest radiograph (Fig. 1b) showed areas of bilateral ground-glass opacity in almost all her lung fields. A chest CT scan (Fig. 2b) revealed bilateral, peripheral, subpleural peribronchovascular consolidation in almost all her lung lobes. Sputum, urine, and blood cultures yielded negative results. Four days after she was treated with loxoprofen for recurrent high fever, we performed bronchoalveolar lavage (BAL) from the left B^4^. The total cell count and lymphocytes in her BAL fluid were elevated to 5.0×10^5^/mL and 38 %, respectively. The result of a drug lymphocyte stimulation test (DLST) in peripheral blood was strongly positive (stimulation index, 330 %) for loxoprofen and negative for ceftriaxone and levofloxacin. The patient was diagnosed with loxoprofen-induced pneumonia. Her respiratory status rapidly worsened after BAL; therefore, she was treated immediately with high-dose methylprednisolone therapy (1 g/day for 3 days) for acute respiratory failure. Her respiratory status and chest radiography findings improved dramatically after initiation of steroid therapy (Fig. 1c). The patient had a Naranjo adverse drug reaction (ADR) probability score of 6 (Table 1), so her symptoms were classified as a probable ADR [1].

**Table 1:** Naranjo adverse drug reaction probability scale calculated for our case

	Question	Response	Our case
1	Are there previous conclusive reports on this reaction?	Yes	+1
2	Did the adverse event appear after the suspected drug was given?	Yes	+2
3	Did the adverse reaction improve when the drug was discontinued or a specific antagonist was given?	No	0
4	Did the adverse reaction appear when the drug was re-administered?	Do not know	0
5	Are there alternative causes (other than the drug) that could have caused the reaction?	Yes	+2
6	Did the reaction reappear when a placebo was given?	Do not know	0
7	Was the drug detected in any body fluid in toxic concentrations?	Do not know	0
8	Was the reaction more severe when the dose was increased/increasing or less severe when the dose was decreased?	No	0
9	Did the patient have a similar reaction to the same or similar drugs in any previous exposure?	Yes	+1
	Total		6

Scoring: >9, definite ADR; 5–8, probable ADR; 1–4, possible ADR; 0, doubtful ADR. *ADR* adverse drug reaction

## Discussion

To the best of our knowledge, this is the first English-language case report of loxoprofen-induced pneumonia. There have been a few case reports of NSAID-induced pneumonia and eosinophilic pneumonia in Japan. These reports include cases of loxoprofen-induced hypersensitivity pneumonia [2] and loxoprofen-induced eosinophilic pneumonia [3]. However, there is no report on the incidence of NSAID-induced pneumonia. In general, the incidence of drug-induced lung disease (DILD) is higher in Japanese than in white patients. For example, in the case of gefitinib, which is the major epidermal growth factor receptor tyrosine kinase inhibitor for non-small cell lung cancer, the incidence of DILD is approximately 2.0 % in Japan, which is higher than that in the USA (0.3 %) [4]. Meanwhile, in the field of idiopathic pulmonary fibrosis (IPF), it has been revealed that some genetic backgrounds, such as telomere length, telomerase mutation, and *MUC5B* promoter polymorphism, are associated with the development of fibrosis in the white population [5]. However, it has been reported that the *MUC5B* promoter polymorphism frequency in Japanese patients with IPF is significantly lower than that in German patients with IPF [6]. These results suggest that other genetic backgrounds may be present in the Japanese population. More recently, research focus on *MUC4* promoter polymorphism indicated an association with the development of DILD in Japanese patients, and that the *MUC4* promoter polymorphism may be associated with ethnic differences in the incidence of DILD. These ethnic differences in promoter polymorphism may account for the difference in incidence of DILD, including that for loxoprofen.

In Japan, most pulmonologists diagnose patients with DILD by the criteria developed by the Japanese Respiratory Society. These criteria suggest that the diagnosis of DILD should be based on the history of treatment with the suspected drug, absence of another disease (for example, infection or pulmonary edema), improvement after withdrawal of the suspected drug, and deterioration after retreatment with the suspected drug [7]. In addition, in Japan, a positive DLST is considered significant evidence of DILD. We diagnosed this patient with loxoprofen-induced pneumonia because of the development of pneumonia after accidental retreatment with loxoprofen, a treatment history of loxoprofen, and exclusion of infection and pulmonary edema. Furthermore, our patient’s peripheral blood DLST for loxoprofen was strongly positive [8].

Her chest CT findings included peripheral, subpleural peribronchovascular consolidation in both lungs in accordance with an organized pneumonia pattern [7, 9]. Although we were able to obtain BAL fluid for analysis, we could not perform transbronchial lung biopsy because of severe coughing during bronchoscopy. However, we confirmed increased cell numbers and lymphocytes in her BAL fluid. Subsequently, steroid pulse therapy was administered because progression of respiratory failure precluded further examination procedures.

## Conclusions

Here we report the first case of loxoprofen-induced pneumonia in the general population. Despite an unknown incidence rate, clinicians should be aware of the potential development of DILD after treatment with loxoprofen.

## Abbreviations

ADR: adverse drug reaction
BAL: bronchoalveolar lavage
CRP: C-reactive protein
CT: computed tomography
DILD: drug-induced lung disease
DLST: drug lymphocyte stimulation test
IPF: idiopathic pulmonary fibrosis
KL-6: Krebs von den Lungen-6
LDH: lactate dehydrogenase
NSAIDs: nonsteroidal anti-inflammatory drugs
PaCO_2_: partial pressure of carbon dioxide in arterial blood
PaO_2_: partial pressure of oxygen in arterial blood
SP-D: surfactant protein-D
SpO_2_: oxygen saturation
WBCs: white blood cells
